# Characterization of RNA silencing components in the plant pathogenic fungus *Fusarium graminearum*

**DOI:** 10.1038/srep12500

**Published:** 2015-07-27

**Authors:** Yun Chen, Qixun Gao, Mengmeng Huang, Ye Liu, Zunyong Liu, Xin Liu, Zhonghua Ma

**Affiliations:** 1 Institute of Biotechnology, Zhejiang University, Hangzhou, 310058, China

## Abstract

The RNA interference (RNAi) plays a critical role in gene regulation in a variety of eukaryotic organisms. However, the role of RNAi remains largely unclear in plant pathogenic fungi. In this study, we explored the roles of core components of the RNAi pathway in *Fusarium graminearum*, the major causal agent of wheat head blight. Our results demonstrated that the hairpin RNA (hpRNA) can efficiently silence the expression level of target gene, and the argonaute protein FgAgo1 and dicer protein FgDicer2 are important in this silencing process. RNAi machinery was not involved in growth, abiotic stress and pathogenesis in *F. graminearum* under tested conditions. We firstly applied high-throughput sequencing technology to elucidate small RNA (17–40 nucleotides) (sRNA) transcriptome in *F. graminearum*, and found that a total of forty-nine micro-like-RNA (milRNA) candidates were identified in the wild-type and ∆FgDICER2, and twenty-four of them were FgDicer2-dependent. Fg-milRNA-4 negatively regulated expression of its target gene. Taken together, our results indicated that the hpRNA-induced gene silencing was a valuable genetic tool for exploring gene function in *F. graminearum*. FgAgo1 and FgDicer2 proteins played a critical role in the hpRNA mediated gene silencing process. In addition, FgDicer2 was involved in sRNA transcription and milRNA generation in this fungus.

RNA silencing or RNA interference (RNAi) is a gene regulatory system through double-stranded RNA (dsRNA) mediated homology-dependent gene inactivation in eukaryotic organisms^1^,^2^,^3^. In this process, the precursor dsRNA are cleaved by a RNaseIII-like enzyme Dicer into small non-coding RNAs (sRNAs) of approximately 19–40 nucleotides (nt). Double stranded sRNAs are subsequently incorporated into a RNA-induced silencing complex (RISC) in which argonaute is the core component and functions as a sRNA-guided endonuclease. The activated RISC unwinds the sRNA in an ATP-dependent reaction, thereby generating an antisense (or guide) strand that targets complementary mRNA transcripts via base-pairing interactions. Subsequent degradation of the targeted mRNA causes inhibition of protein biosynthesis^4^,^5^. RNA-dependent RNA polymerase (RdRp) amplifies the sRNA signal by generating dsRNA from single-stranded transcripts either by *de novo*, primer-independent second-strand synthesis or by using sRNAs as primers to synthesize RNA complementary to the target mRNA^2^.The RNAi-related phenomenon called “quelling” in fungi was first described in the ascomycete *Neurospora crassa*^6^. It has been shown that the quelling pathway participates in various development and physiological processes, including the gene regulation, genomic stability, heterochromatin formation, invasive nucleic acids defense, and gene regulation in several fungal species^3^,^7^. Although the core RNAi components exist in many fungi, their physiological roles are various and poorly understood.

MicroRNAs (miRNAs) or miRNA-like small RNAs (milRNAs) are small non-coding endogenous RNAs of approximately 22 nt, and have been well investigated in plant and animals. They negatively regulate gene expression *via* targeting mRNAs by complementary binding to the open-reading frame or un-translated (UTR) regions of specific target genes in animals and plants^8^. In animals, miRNAs have been shown to play various roles in cell development, proliferation and differentiation, apoptosis, carcinogenesis, and immunity^9^,^10^,^11^. In plants, they are also involved in plant development, stress response and antibacterial resistance^5^,^12^,^13^. More recently, milRNAs have also been identified in several fungal species, including *N. crassa*, *Sclerotinia sclerotiorum*, *Metarhizium anisopliae*, *Cryptococcus neoformans*, and *Penicillium marneffei*^2^,^14^,^15^,^16^,^17^. However, the potential roles of milRNAs in plant pathogens have not been documented.

*Fusarium graminearum* (sexual state *Gibberella zeae*) is a destructive pathogenic fungus causing Fusarium head blight (FHB) of wheat, barley and other cereals^18^,^19^. The disease can result in severe yield losses. In addition, infested grains contaminated with mycotoxins, such as deoxynivalenol (DON), nivalenol and zearalenones, are harmful to humans and animals^20^,^21^. Despite the high economic impact of FHB, efficient strategies for the management of FHB have not been well developed. Application of systemic fungicides, such as sterol demethylation inhibitors (DMIs), is a commonly-used approach for controlling FHB. However, the emergence of DMI-resistant *F. graminearum* over the last few years further accelerates the need for alternative control strategies^22^. Host induced gene silencing (HIGS) shows a potential approach for the development of resistant plant cultivars against *Fusarium* spp.^23^,^24^, which silences target-gene transcription via RNAi pathway in *F. graminearum* by the dsRNA generated in transgenic plant. But currently the biological function and core components role of the RNAi pathway has not been documented in *F. graminearum* and other *Fusarium* spp. In this study, we aimed to characterize the role of RNAi pathway core components in *F. graminearum*. Our results demonstrated that the hpRNA can efficiently reduce mRNA transcription levels of target genes, and the argonaute protein FgAgo1 and dicer protein FgDicer2 are important in this silencing process. In addition, we also observed that RNAi machinery is not involved in the growth, abiotic stress and pathogenesis under tested conditions. Furthermore, we found that FgDicer2 plays a role in sRNA transcription and micro-like RNA generation.

## Results

### Hairpin RNA (hpRNA) is able to induce endogenous genes silencing in *F. graminearum*

**Figure 1:**
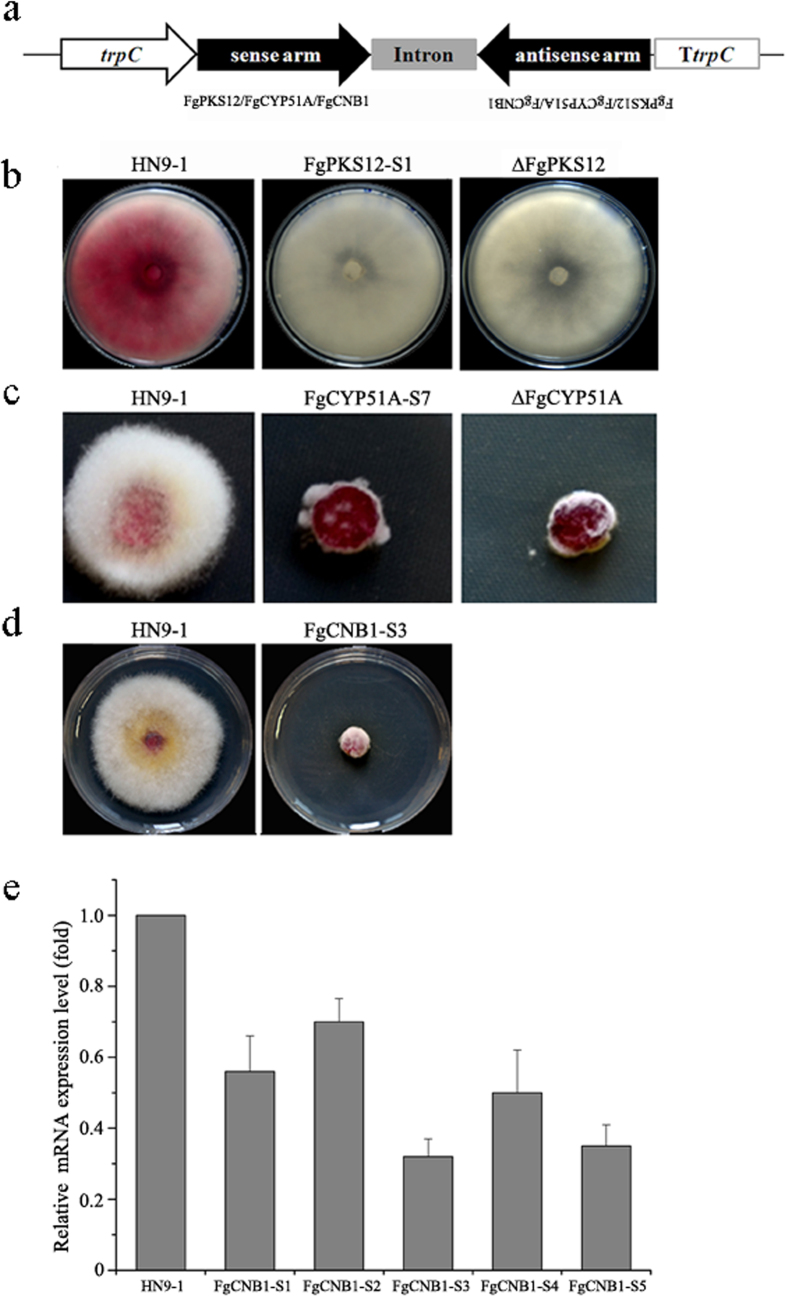
Phenotypic analyses of hpRNA-induced single gene silencing in *F. graminearum.* (**a**) Schematic representation of hpRNA-expressing constructs. The hairpin inverted-repeat targeted region of *FgPKS12, FgCYP51A,* or *FgCNB1* was obtained by PCR and inserted into the multiple cloning site of hpRNA-induced gene silencing vector pSilent-1(**b**) Colony morphology of the RNAi-silenced *FgPKS12* transformant. The wild-type HN9-1 and *FgPKS12* gene silencing transformant were grown on PDA for 7 days at 25 °C. The silencing mutant FgPKS12-S1 displayed white colony, similar to the *FgPKS12* deletion mutant (∆FgPKS12). (**c**) Susceptibility of *FgCYP51A* silencing transformant to the DMI fungicide triadimefon. *FgCYP51A* silencing transformant FgCYP51A-S7 were grown on PDA supplemented with triadimefon (5 μg mL^−1^) for 3 days at 25 °C. Similar to the *FgCYP51A* deletion mutant (∆FgCYP51A), the silencing transformant showed increased sensitivity to triadimefon. (**d**) Growth pattern of the essential gene *FgCNB1* silencing transformant. The *FgCNB1* silencing transformant FgCNB1-S3 and the wild-type HN9-1 were cultured on PDA at 25 °C. The image was taken after 3 days of incubation. (**e**) The relative mRNA expression level of *FgCNB1* in silencing transformants in comparison to that in the wild type. Line bars denote standard errors of three repeated experiments.

To examine RNAi in *F. graminearum*, we chose two genes *FgPKS12* and *FgCYP51A* that would result in clear phenotypes if hpRNA-induced gene silencing is successful. The disruption mutant of *FgPKS12,* which is involved in pink pigment biosynthesis, showed white colony morphology on potato dextrose agar (PDA) (Fig. 1b). *FgCYP51A* encodes a 14-α demethylase, and our previous study showed that the deletion mutant of *FgCYP51A* (∆FgCYP51A) exhibited increased sensitivity to sterol demethylation inhibitor (DMI) fungicides^25^. pSilent-1, a hpRNA-induced gene silencing vector^26^, was used to produce hpRNA in *F. graminearum.* pSilent-PKS12 and -CYP51A for generating hpRNA of a *FgPKS12* fragment (426 bp) and *FgCYP51A* (401 bp) respectively were constructed via the insertion of the two-step polymerase chain reaction (PCR) products into the multiple cloning sites (Fig. 1a), and subsequently introduced into the *F. graminearum* wild-type strain HN9-1.

Phenotypic analysis of 58 transformants containing the pSilent-PKS12 obtained from five independent transformation events showed that 11 (19.0%) transformants displayed white colony morphologies after a 7-day incubation on PDA. The colony patterns of these 11 transformants were similar to that of ∆*FgPKS12,* whereas the wild-type HN9-1 produced obviously red pigment (Fig. 1b). For *FgCYP51A,* 12 out of 61 transformants (19.7%) were similar to ∆FgCYP51A in sensitivity to the DMI fungicide triadimefon (Fig. 1c), indicating that *FgCYP51A* had been silenced in these 12 transformants.

To examine whether this silencing tool works for essential genes in this organism, we chose a house-keeping gene *FgCNB1* (FGSG_06103) (the calcineurin regulatory subunit B gene) as the target gene in a silencing test. After the pSilent-FgCNB1 silencing plasmid was introduced into HN9-1, 5 out of 49 (10.2%) transformants showed severe growth defects (Fig. 1d). The mRNA levels of *FgCNB1* in these FgCNB1 silencing transformants were decreased to 30%–70% in comparison to that in the wild type (Fig. 1e). These results suggested that *FgCNB1* was silenced effectively in these transformants.

**Figure 2:**
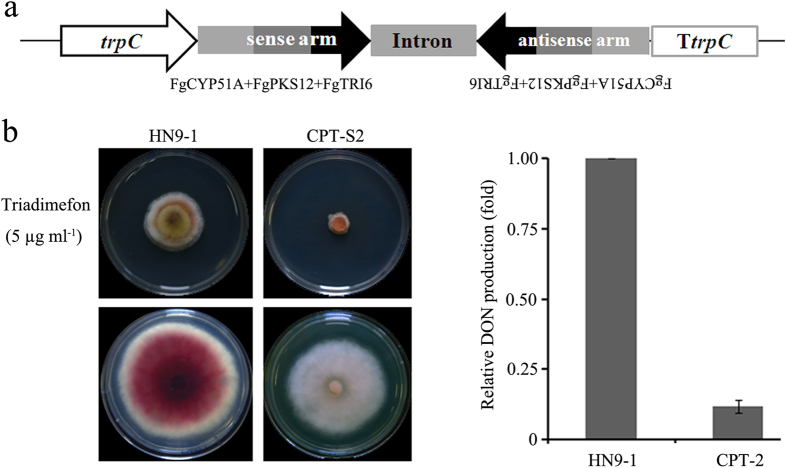
Simultaneous silencing of multiple genes in *F. graminearum* via expression of hpRNA with chimeric inverted repeats. (**a**) Schematic representation of the chimeric inverted-repeat fragment construction. The target fragment of *FgCYP51A, FgPKS12,* and the DON biosynthesis gene *FgTRI6* was constructed using the joint PCR strategy and then inserted into the multiple cloning site of pSilent-1. (**b**) Phenotypic analyses of the silencing transformant. The silencing transformant CPT-S2 showed increased susceptibility to triadimefon (5 μg mL^−1^; left top), displayed white colony (left bottom), and produced drmatically less DON than the wild type HN9-1 (right panel).

In addition to individual genes, we also examined whether multiple genes could be silenced simultaneously using a chimeric hairpin vector (Fig. 2a). We constructed a tandem DNA fragment including *FgCYP51A, FgPKS12,* and *FgTri6* (a mycotoxin deoxynivalenol [DON] biosynthesis gene) with the joint PCR strategy and then inserted the fragment into the pSilent-1 vector to obtain a recombination plasmid, designated pSilent-CPT. After transformation of the wild type with pSilent-CPT, 10 out of 50 transformants showed multi-phenotypic changes, including white colony, increased sensitivity to triadimefon, and reduced DON production (Fig. 2b). The segregated silencing phenomenon was not observed, indicating that the triple genes were silenced simultaneously in these transformants. In addition, PCR assays showed that the open reading fragments (ORFs) of target genes were intact in all gene-silencing transformants (data not shown), suggesting that this phenotypic variations were not caused by ORF disruption. Taken together, these results clearly indicate that the RNAi pathway exists in *F. graminearum*, and hpRNA generated with the pSilent-1-based vector can induce the silencing of endogenous genes, including nonessential and essential genes.

### The *F. graminearum* genome contains major components of the RNAi pathway

We searched the *F. graminearum* genome ( http://www.broadinstitute.org/annotation/genome/fusarium_group/GenomesIndex.html) for RNAi components and identified two dicer proteins (FgDicer1 and FgDicer2), two argonaute proteins (FgAgo1 and FgAgo2), and five RNA-dependent RNA polymerases (FgRdRp1–5). All FgRdRp proteins contain the RdRp domain, which is highly conserved in fungi (Fig. S1a). FgRdRp2 (FGSG_08716) also contained an additional RNA recognition motif (RRM) in the N-terminal region that is present in a minority of RdRp enzymes. In addition to the RdRp domain, the putative FgRdRp5 protein (FGSG_09076) harbors an EAD (DEAD-like helicase) and an AAA domain at the C-terminus (Fig. S1a). Both FgAgo1 and FgAgo2 proteins contain three domains (DUF1785, PAZ, and Piwi), which have been identified in other argonaute orthologs. PAZ functions as a nucleic acid binding domain, with a strong preference for single-stranded nucleic acids (RNA or DNA) or RNA duplexes with single-stranded 3’ overhangs^27^. The function of Piwi domain is dsRNA-guided hydrolysis of ssRNA and Dicer binding^28^. FgDicer1 and FgDicer2 contain the un-canonical Dicer protein structures with a DEAD box, a helicase C domain (hel C), a Dicer dimerization domain, and two RNase III domains (RNase IIIa and RNase IIIb; Fig. S1a). In addition, FgDicer2 also contains a double-stranded RNA binding motif (DSRM; Fig. S1a).

To explore the molecular evolution of the RNA silencing machinery in fungi, we performed phylogenetic analyses of RdRp-, argonaute-, and dicer-like proteins from representative fungal species. *Arabidopsis thaliana* was used as an outgroup member. The five FgRdRp proteins were clustered into four groups. FgRdRp1 and FgRdRp4 were closely related to *N. crassa* qde1 with 39% and 32% identity, respectively. FgRdRp3 was homologous to *N. crassa* rrp3 with 38% identity, whereas FgRdRp2 was more closely related to *Magnaporthe oryzae* RdRp2. It is interesting that FgRdRp5 was more closely related to plant RdRp-like protein (Fig. S1b). These results indicate that FgRdRp orthologs in *F. graminearum* may have evolved from different ancestors. The FgAgo1 protein is closely related to the canonical fungal argonaute protein *N. crassa* Qde2 (with 36% identity), whereas FgAgo2 showed high similarity to *N. crassa* Sms2 (with 43% identity), which was involved in meiotic silencing through the unpaired DNA (MSUD) pathway^29^ (Fig. S1b). Similar to the argonaute protein distribution, FgDicer2 and *N. crassa* Dicer2 were clustered within a single group, and FgDicer1 was closely related to *N. crassa* Dicer1, which functions in the MSUD pathway^30^ (Fig. S1b). These phylogenetic trees indicated that FgAgo1 and FgDicer2 may play a role in the RNAi pathway, whereas the combination of FgAgo2 and FgDicer1 might be required for the MSUD pathway in *F. graminearum.*

### RNAi machinery is not involved in vegetative development and pathogenesis in *F. graminearum* under tested conditions

Previous studies have shown that the RNAi pathway plays a role in the development and physiology of several fungi^2^,^3^,^7^,^31^,^32^,^33^,^34^. Thus, we characterized the physiological and cellular functions of RNAi components in *F. graminearum* by constructing deletion mutants of each gene using a homologous recombination strategy. For further functional analysis of argonaute and dicer proteins, we constructed the *FgAGO1* and *FgAGO2* double deletion mutant (thereafter named ΔFgAGO12). Concurrently, the double mutant of ΔFgDICER12 was also constructed via deletion of *FgDicer1* in ΔFgDICER2. All mutants were identified using PCR and further confirmed by Southern blotting assays (Fig. S2).

As shown in Fig. S3a, each mutant did not show detectable changes in mycelial growth or colony morphology on PDA. The pathogenicity assay showed that all mutants caused the typical scab symptoms on flowering wheat head and the rot symptoms on tomato, similar to the wild type (Fig. S3b). Meanwhile, those mutants showed normal asexual and sexual development (data not shown). In addition, we examined the role of RNAi components in response to various environmental stress conditions, including the DNA damage agents methyl methanesulfonate, hydroxyurea, and histidine; the osmotic agents NaCl, KCl, glucose, and sorbitol; the cell wall stress agents Congo red and caffeine; and the divalent cations Mg^2+^ and Ca^2+^. All mutants displayed similar susceptibility to tested stress agents in comparison to the wild type (Fig. S3c). Meanwhile, we also constructed the double gene mutants of *FgRDRPs* (10 combinations) and tested all above phenotypes of them. There were no visible changes, compared to the wild type (data not shown). These data suggest that the RNAi components are not involved in growth, pathogenicity, or multiple stress responses in *F. graminearum* at least under tested conditions.

### FgAgo1 and FgDicer2 play important roles in hpRNA-induced gene silencing

**Figure 3:**
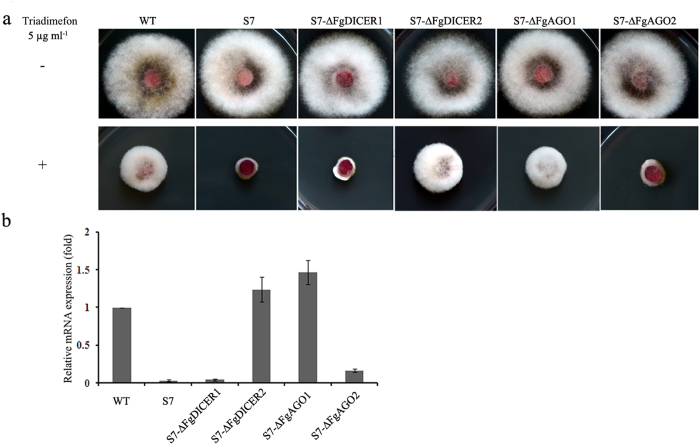
The role of argonautes and dicers in hpRNA-induced gene silencing in *F. graminearum*. (**a**) Growth pattern of HN9-1, *FgCYP51A* gene silencing transformant FgCYP51A-S7, argonaute gene mutants in the FgCYP51A-S7 background (S7-∆FgAGO1 and S7-∆ FgAGO 2), and dicer gene mutants in the FgCYP51A-S7 background (S7-∆FgDICER1 and S7-∆FgDICER2) on PDA with or without DMI fungicide triadimefon (5 μg mL^−1^). The plate photographs were taken after 3 days of incubation at 25 °C. (**b**) The relative mRNA expression levels of *FgCYP51A* in the wild type, silencing transformant FgCYP51A-S7, S7-∆FgDICER1, S7-∆FgDICER2, S7-∆FgAGO1, and S7-∆FgAGO2 treated with triadimefon. Mycelia were collected after each strain was treated with 5 μg mL^−1^ triadimefon for 6 h, then the RNA extracted from each strain was used for quantitative RT-PCR assays. Expression of the actin gene was used as the reference. Line bars denote standard errors of three repeated experiments.

As described previously, RNAi occurs in *F. graminearum* (Fig. 1, Fig. 2). Therefore, we were interested in investigating the role of each component in RNAi. Because five predicted RdRp proteins in the *F. graminearum* genome may function redundantly, it is difficult to characterize the potential role of RdRp proteins. Thus, we focused on the characterization of FgAgo1/2 and FgDicer1/2 in this study. The transformant FgCYP51A-S7, in which *FgCYP51A* has been silenced, was stable in triadimefon sensitivity (Fig. 1c). Quantitative RT-PCR confirmed that *FgCYP51A* gene expression was reduced by approximately 96% in FgCYP51A-S7 in comparison to the wild type after treatment with triadimefon (Fig. 3b). Hence, we constructed the argonaute and dicer gene deletion mutants using FgCYP51A-S7 as the parental strain, and the resulting mutants (S7-∆FgAGO1, S7-∆FgAGO2, S7-∆FgDICER1, and S7-∆FgDICER2) were tested for triadimefon susceptibility. As shown in Fig. 3a, S7-∆FgAGO1 and S7-∆FgDICER2 completely restored the resistance to triadimefon, and were identical to the wild type in colony morphology. The mRNA expression levels of *FgCYP51A* in these mutants were not significantly different to the wild-type level (Fig. 3b), indicating that hpRNA-induced gene silencing was completely blocked in S7-∆FgAGO1 and S7-∆FgDICER2. In contrast, S7-∆FgAGO2 and S7-∆FgDICER1 did not change their susceptibility to triadimefon (Fig. 3a). Quantitative RT-PCR experiments also confirmed that expression of *FgCYP51A* remained a low level in both mutants, similar to FgCYP51A-S7 (Fig. 3b). Taken together, these results indicate that FgAgo1 and FgDicer2 play a major role in hpRNA-induced gene silencing in *F. graminearum.*

### Different mRNA expression patterns of argonaute and dicer genes in conidia and mycelia

**Figure 4:**
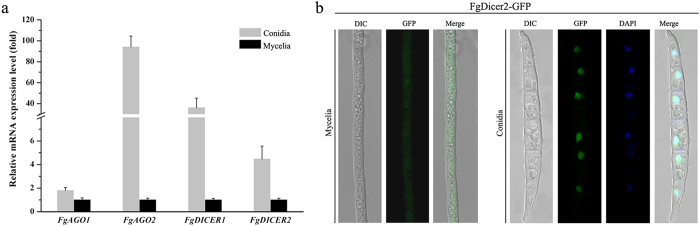
Relative mRNA expression patterns of argonaute and dicer genes. (**a**) The relative mRNA expression level of argonaute or dicer genes in conidia represents the relative amount of each gene mRNA in the mycelia determined by quantitative RT-PCR. Line bars indicate standard errors from three repeated experiments. (**b**) Localization of the FgDicer2-GFP fusion protein in mycelia and conidia. Fresh mycelia and conidia were collected and visualized with a Zeiss LSM780 confocal microscope for GFP signals. To observe nuclei, fresh mycelia or conidia were washed with sterilized water and stained with 10 μg mL^−1^ 4′6-diamidino-2-phenylindole (DAPI, Sigma).

A previous study showed that mRNA expression levels of argonaute and dicer genes in mycelia were significantly different from those during the yeast phase in the thermal dimorphic fungus *P. marneffei*^35^. Therefore, we also analyzed the mRNA expression patterns of argonaute and dicer genes in conidia and mycelia of *F. graminearum.* Quantitative RT-PCR assays showed that mRNA expression levels of *FgAGO1* and *FgDICER2* in conidia increased by 1.8- and 4.5-fold, respectively, in comparisons to those in mycelia. It is surprising that the mRNA expression levels of *FgAGO2* and *FgDICER1* in conidia were 94- and 36-fold higher than those in mycelia, respectively (Fig. 4). These data indicate that *FgAGO2* and *FgDICER1* may play a role in conidia. However, the conidia morphology of argonaute and dicer mutants did not show significant changes under tested conditions in comparisons with the wild type (data not shown). Furthermore, the localizations of FgAgo1 and FgDicer2 were visualized using confocal laser microscopy. The GFP signals of the FgAgo1-GFP fusion protein were not detectable, suggesting that the amount of FgAgo1 protein was very low under the tested conditions. The FgDicer2-GFP fusion protein was mainly localized to the cytoplasm of aerial hyphae, while concentrated into the nucleus of conidia (Fig. 4), indicating that it may function differently in conidia and mycelia.

### Small RNAs are expressed differentially in the wild type and ∆FgDICER2 mutant

**Figure 5:**
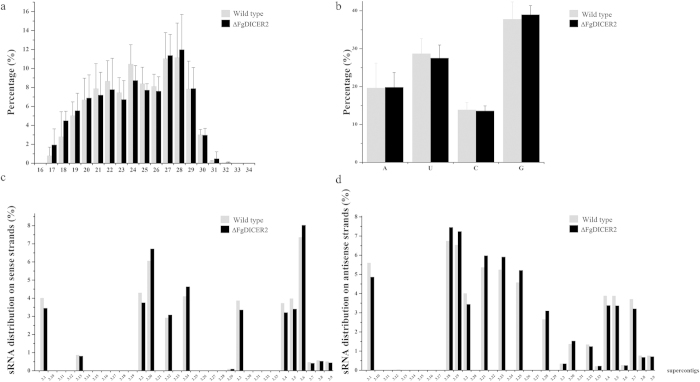
Characterization of small RNAs in the wild-type HN9-1 and ∆FgDICER2 of *F. graminearum.* (**a**) The percentage of different sizes of small RNAs. Line bars indicate standard deviations of three repeated experiments. (**b**) Nucleotide frequency of the 5′ end of small RNAs in mycelia of the wild type and ∆FgDICER2. The percentages of small RNA distributions on the sense (**c**) and antisense (**d**) strands of supercontigs.

Since FgDicer2 is critical for hpRNA-induced gene silencing, we inferred that FgDicer2 may be involved in small RNA (sRNA) biosynthesis in *F. graminearum.* To examine RNAs that may be generated by FgDicer2, we isolated RNAs ranging from 17 to 40 nt and constructed sRNA libraries from both the wild type strain and ∆FgDICER2. After RNA sequencing, a total of 28,325,047 and 28,088,824 clean tags (≥17 bp) were obtained from the wild type and ∆FgDICER2 respectively in three repeated experiments (Table S1). The tags were used for mapping to the *F. graminearum* genome. As indicated in Table S1, average of 89.22 ± 1.51% and 88.98 ± 1.61% total sRNAs from the wild-type strain and ∆FgDICER2, respectively, were mapped perfectly to the reference genome with 100% coverage and 100% identity (Table S1). The sRNAs identified from both the wild-type and ∆FgDICER2 strains were 17–32 nt in length, with the peak at 27–28 nt. In addition, 37.79% and 38.99% sRNAs from the wild type and ∆FgDICER2 respectively had a preference for 5’G (Fig. 5a,b). This differs from what has been observed in animals^36^ and plants^37^, in which sRNAs have a strong preference for 5’U . As expected, the unique sRNAs in the wild type differed significantly from those in ∆FgDICER2. Only 15.87% of unique sRNAs were found in both the wild type and ∆FgDICER2 strains in three repeated experiments (Table 1).

Although small RNAs obtained from the wild type and ∆FgDICER2 were mapped to entire chromosomes, the distributions of sRNAs on different super-contigs vary dramatically in both the wild type and ∆FgDICER2. Moreover, the numbers of sRNAs derived from sense strands were drastically different from those from antisense strands (Fig. 5c,d). As results shown in Fig. 5, the origins of sRNAs in ∆FgDICER2 were different from those of the wild type. These results suggested that FgDicer2 plays an important role in sRNAs biogenesis in *F. graminearum.*

### FgDicer2-dependent biogenesis of milRNA (micro-like-RNA) in *F. graminearum*

Because milRNA mediate gene silencing in fungi^14^,^35^, we explored whether milRNAs are also present in *F. graminearum.* Using the MIREAP program, we identified 49 milRNA candidates (named FgmilRNA1–49) with an average of approximately −58.83 kcal mol^−1^ folding free energies (Table S2) according to RNAfold ( www.tbi.univie.ac.at/~ivo/RNA/RNAfold.html). This was close to those of the phytopathogenic fungus *S. sclerotiorum* and *Arabidopsis* miRNA precursors (−60.9 and −59.5 kcal mol^−1^, respectively) and much lower than the folding free energies of rRNA (33 kcal mol^−1^) or tRNA (27.5 kcal mol^−1^)^16^,^38^. The lengths of these identified milRNAs ranged from 20 to 24 nt, with an average of 21 nt. These milRNA candidates were produced from 93 loci and were distributed on intergenic (64%), exon-sense (26%) and intron-sense (10%) regions.

**Figure 6:**
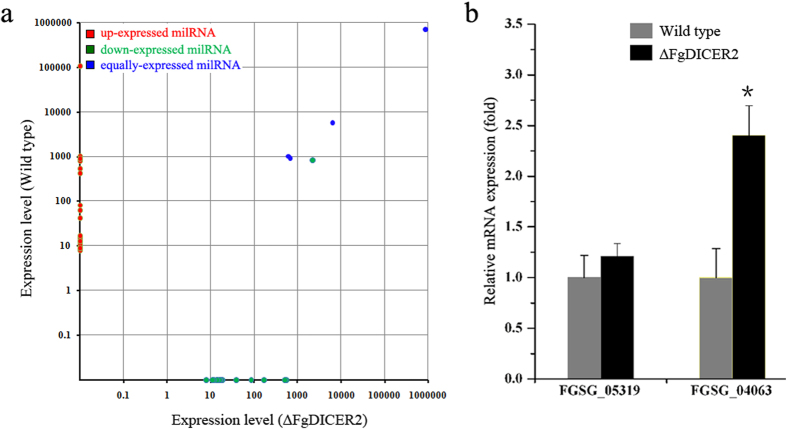
The milRNA expression patterns in *F. graminearum*. (**a**) Comparisons of expression levels of 49 milRNAs candidates in the wild type and ∆FgDICER2. (**b**) Relative mRNA expression level of Fg-milRNA-4 target gene in the wild type and ∆FgDICER2. The relative mRNA expression levels of Fg-milRNA-3 target gene (*FGSG_05319*), Fg-milRNA-4 target gene (*FGSG_04063*) in ∆FgDICER2 represent the relative amount of each mRNA in the wild type determined *via* quantitative RT-PCR. Fg-milRNA-3 target-gene (*FGSG_05319*) was measured as a control. Line bars indicate standard errors from three repeated experiments. The star symptom meant there was significantly different.

The expression of these milRNAs was further analyzed in the wild type and ∆FgDICER2. As shown in Fig. 6a and Table S2, twenty-four milRNAs were not detected in ∆FgDICER2, indicating that these milRNA candidates might be FgDicer2- dependent. The expression levels of Fg-milRNA-1, −3, −23 and −49 did not change significantly in ∆FgDICER2 in comparison to the wild type. It is interesting that twenty-one milRNA candidates were identified from ∆FgDICER2 but not from the wild type. To determine whether *F. graminearum* milRNAs regulated target genes, we examined the mRNA transcription levels of Fg-milRNA-4 target-gene, *FGSG_04063*, in the wild type and ∆FgDICER2. Fg-milRNA-3 target-gene (*FGSG_05319*) was also measured as a control. The mRNA expression level of *FGSG_04063* was upregulated 2.4-fold in ∆FgDICER2 in comparison to those in the wild type (Fig. 6b). These results indicated that FgDicer2 was involved in the milRNA biosynthesis and FgDicer2-dependent Fg-milRNAs could regulate expression of their target genes in *F. graminearum.*

## Discussion

The RNA silencing mechanism has been exploited as a novel and efficient genetic tool for gene function analysis using a hpRNA-expressing plasmid or an opposing-dual promoter system in filamentous fungi. This provides an alternative approach for elucidating the functions of essential genes as their knockout mutants are lethal^7^,^26^. Different hpRNA constructs and opposing-dual promoter–produced sRNA targeting endogenous genes have been tested in several fungal species with varying success^39^,^40^. Meanwhile, sRNA molecules are mobile and can be taken up by fungal cells. The treatment of fungal protoplasts or conidia of *Aspergillus flavus, Aspergillus parasiticus, F. graminearum,* and *Fusarium oxysporum* with siRNAs could reduce the expression of their target genes^23^,^41^,^42^,^43^. Our results indicate that *F. graminearum* has the functional RNA silencing machinery that processes hpRNA into sRNAs, and the resulting sRNAs further target homologous mRNAs for cleavage. The introduction of hpRNA into *F. graminearum* can decrease the expression of single and multiple genes simultaneously. Therefore, hpRNA-induced gene silencing would be a useful tool for functional analysis of essential or functionally redundant genes in *F. graminearum.*

It is important to note that the frequencies of RNA-mediated gene silencing with the pSilent-1-based vectors were only about 10%–20% in *F. graminearum*. In *Venturia inaequalis,* however, agrobacterium-mediated transformation of T-DNA containing inverted repeats of target gene(s) could silence the target genes in more than 50% of transformants^44^. In addition, more than 30% of transformants containing RNA silencing vector pSD1 and pSilent-1 showed significantly decreased expression levels of target mRNAs in *M. oryzae*^26^,^45^. In *Schizosaccharomyces pombe,* hpRNA can activate two independent gene silencing pathways, so named heterochromatin formation (transcriptional gene silencing) and post-transcriptional gene silencing, depending on the location of the targeted gene within the host genome^46^,^47^,^48^. It is possible that hpRNA also induces transcriptional silencing in *F. graminearum,* which can target the hpRNA transgene to cause transcriptional self-silencing and subsequently reduce the transcription level of hpRNA transgene. Meanwhile, the vector construction and promoters for generating hpRNA are also involved in the silencing efficacy^45^,^49^. Therefore, more suitable vectors are still required for the high-efficiency hpRNA-mediated gene silencing in *F. graminearum.*

Phenotypic analyses of the mutants of RNAi pathway components in a few filamentous fungi and yeasts suggest that although RNAi may play a role in development and physiology, its functions may vary significantly in different fungal species. In this study, we characterized components of the RNAi machinery in *F. graminearum,* including RdRP1–5, Argonaute 1/2, and Dicer 1/2 using the gene deletion strategy. No phenotypic alternations were observed in these mutants under tested conditions, which is in agreement with the finding from previous studies on *A. nidulans*^50^. In *C. neoformans*, the components of RNAi pathway are involved in regulating growth, conidiation, and genome protection against exogenous nucleic acids, such as virus and transposons^2^,^3^,^7^,^31^,^33^. The RNAi pathway also plays an important role in controlling transposon activity and genome integrity during vegetative growth in *N. crassa, Mucor circinelloides,* and *M. oryzae*^33^,^51^,^52^,^53^. In *Trichoderma atroviride,* Dcr2 and Rdr3 control conidium development. Furthermore, Dcr1, together with Dcr2, controls vegetative growth, as the Δdcr1Δdcr2 mutant exhibits reduced growth^2^. In addition, the mutants of ∆dcl2 and ∆agl2 of *C. parasitica* were hypersensitive to virus^31^,^54^. Transcriptional analysis of RNAi mutants of *C. neoformans* and *Saccharomyces castellii* showed increased activity of transposons and accumulation of siRNA precursors^32^,^33^,^55^. In the fission yeast *S. pombe,* RNA silencing components are involved in sequence-specific heterochromatin formation via histone modifications and also regulate cell cycle progression and govern cell survival against DNA damage agents^56^,^57^. These results indicate that the functions of RNAi components vary significantly among different fungal species.

In the current study, we found that the deletion of FgDicer2 or FgAgo1 in hpRNA-mediated gene silencing transformants restored the mRNA expression level of target genes to the wild-type level (Fig. 3). Meanwhile, phylogenetic analyses revealed that FgAgo1 is related to *N. crassa* qde2, a canonical argonaute protein (Fig. S1b). In addition, FgDicer2 was clustered together with the dicer2 of *N. crassa,* which is an RNaseIII-like enzyme critical for the RNAi pathway. Based on these data, we generated a model of the participation of such RNAi components in responsible for hpRNA, premiRNA, or exogenous dsRNA in *F. graminearum* (Fig. S4). Small RNA precursors, such as dsRNA or hpRNA, are processed primarily by FgDicer2 in mycelium. The siRNAs/ex-siRNAs are loaded onto FgAgo1 and target the cognate mRNA or genome locus for gene silencing. Amplification of the sRNA pool may be associated with RdRP proteins.

In this study, we also performed a detailed analysis of the sRNA transcriptome (17–40 nucleotides in length) in a *Fusarium* species and found that *F. graminearum* possesses a diverse set of sRNAs and displays similar patterns as the plant pathogen *M. oryzae* and the biocontrol agent *T. atroviride*^2^,^53^. In addition, a total of 24 milRNAs dependent on FgDicer2 were identified from sRNA pools in this study. The milRNAs have been identified from filamentous fungi *N. crassa*^14^, *M. anisopliae*^17^, and *P. marneffei*^35^ as well as the plant pathogenic fungus *S. sclerotiorum*^16^, and they play an important role in regulating gene expression in these fungi. It is reasonable to hypothesize that milRNAs may also regulate gene expression in *F. graminearum.* In *N. crassa*, milRNAs are produced by at least four different mechanisms that use a distinct combination of factors, including dicers, QDE-2, the exonuclease QIP, and a RNase III domain-containing protein MRPL3^14^. Using *N. crassa* QIP and MRPL3 as queries, we identified the homologous loci FGSG_06722 and FGSG_01970 from the *F. graminearum* genome. Thus, further studies are required to explore the biological roles of these proteins in milRNA biosynthesis and milRNAs in *F. graminearum.*

## Methods

### Strains and culture conditions

The *F. graminearum* wild-type strain HN9-1 collected from Henan Province, China was used as a parental strain for transformation experiments. The wild type and resulting transformants in this study were grown on potato dextrose agar (PDA) (potato 200 g, glucose 20 g, agar 15  g, and 1 L water) for mycelial growth tests and examination of colony characteristics. For conidiation assays, fresh mycelia (50 mg) of each strain taken from the periphery of a 3-day-old colony were inoculated in a 50 ml flask containing 20 ml of carboxymethyl cellulose (CMC) medium (NH_4_NO_3_ 1 g, KH_2_PO_3_ 1 g, MgSO_4_.7H_2_O 0.5 g, yeast extract 1 g, carboxymethyl cellulose 15 g, and 1 L water). The flasks were incubated at 25 °C for 4 days in a shaker (180 rpm). For each strain, the number of conidia in the broth was determined using a hemacytometer. The experiment was repeated three times independently.

For determination of fungal growth under environmental stress conditions, serial dilutions of conidial suspension of each strain were spotted on minimal medium (MM) (10 mM K_2_HPO_4_, 10 mM KH_2_PO_4_, 4 mM (NH_4_)_2_SO_4_, 2.5 mM NaCl, 2 mM MgSO_4_, 0.45 mM CaCl_2_, 9 mM FeSO_4_, 10 mM glucose, and 1 L water, pH 6.9) supplemented with the following compounds: DNA damage agents methyl methanesulfonate, hydroxyurea and histinde, the osmotic stress agents NaCl, KCl, glucose, and sorbitol, cell wall stress agents Congo red and caffeine, or divalent cation Mg^2+^ and Ca^2+^ at various concentrations (indicated in the Figure legends). After the plates were incubated at 25 °C for 2 days, the colony morphology of each strain was imaged. Each experiment was repeated three times independently.

### Generation of gene deletion mutants

For *FgRDRP1* gene, the gene disruption vector pBS-*FgRDRP1*-Del was constructed by inserting two flanking fragments of *FgRDRP1* gene into two sides of *HPH* (hygromycin resistance gene) in the pBS-HPH1 vector^58^. The upstream flanking fragment of *FgRDRP1* was amplified from the wild-type DNA using the primer pair *FgRDRP1-*KO1/-KO2 (Table S3). The resulting 695 bp fragment was inserted into *Xho* I-*Sal* І sites of the pBS-HPH1 vector to generate the plasmid pBS-*FgRdRp1*-up. Subsequently, a 945 bp downstream flanking fragment of *FgRDRP1* was amplified from the wild-type DNA using the primer pair *FgRdRp1-*KO3/-KO4 (Table S3) and was inserted into the Hind III-*Bam*H I site of pBS-*FgRDRP1*-up vector to generate the plasmid pBS-*FgRDRP1*-Del. The plasmid was further transformed into protoplasts of the progenitor HN9-1 using the previously published protocol^59^,^60^. Hygromycin was added to a final concentration of 100 μg mL^−1^ for selection of transformants, and putative *FgRDRP1* deletion mutants were identified by PCR assays with the primer pair *FgRDRP1-*ID1/-ID2 (Table S3) and were further verified by Southern hybridization assays. Using the same strategy, the *FgRDRP2, FgRDRP3, FgRDRP4, FgRDRP5, FgAGO1, FgAGO2, FgDICER1* and *FgDICER2* gene single deletion mutants were constructed.

In order to generate the double mutant of *FgAGO1* and *FgAGO2*, *FgAGO2* was knocked out in the *FgAGO1* deletion mutant (ΔFgAGO1). A geneticin resistance gene cassette (*NEO*) was amplified from pCA-NEO and replaced the hygromycin region in the vector pBS-*FgAGO2*-Del at the Sal I-*Bam*H I site. The protoplast transformation of ΔFgAGO2 with the recombination plasmid pBS-*FgAGO1*-NEO-Del was conducted using the method as described above except that geneticin was used as a selection agent. Using the same strategy, the double mutant of *FgDICER1* and *FgDICER2*, and double mutants of *FgRDRP* genes were also generated.

To analyze the gene silencing efficacy in RNAi component mutants, *FgAGO1, FgAGO2, FgDICER1* and *FgDICER2* were knockout individually in FgCYP51A-S7 silencing transformant with geneticin as a selected marker. All of the mutants generated in this study were preserved in 15% glycerol at −80 °C.

### Construction of hairpin RNA silencing vectors

To silence *FgCYP51A* expression in the wild type, a 400 bp gene fragment was amplified with the primer pair *FgCYP51A*-SP1/-SP2 and inserted into the XhoI/HindIII site of pSilent-1.The resulting plasmid was named pSilent-FgCYP51AS-up. The same fragment of *FgCYP51A* was amplified with the primer pair FgCYP51A-SP3/-SP4, and inserted into the KpnI/Bgl II site of pSilent-FgCYP51AS-up. The recombined silencing plasmid was designated as pSilent-FgCYP51AS and transferred into the wild-type strain with the PEG-mediated transformation method as described above. The hairpin RNA silencing plasmids for *FgPKS12* and *FgCNB1* were constructed with the same strategy. For constructing a chimeric hairpin RNA silencing vector to silence *FgCYP51A*, *FgPKS12* and *FgTIR6* simultaneously, a fragment from each gene was amplified and the resulting fragments were further fused with overlapping PCR. The fused fragment was purified and inserted into pSilent-1 as described above to generate the silencing vector pSilent-CPT. Primer pairs used to amplify the target regions are listed in Table S3.

### Microscopic examinations of FgAgo1 and FgDicer2 localization

In order to visualize the localization of FgAgo1 and FgDicer2 in mycelia and conidia, the FgAgo1-GFP and FgDicer2-GFP fusion proteins were constructed. Briefly, the fragment of *FgAGO1* containing promoter region and open reading fragment (without stop codon) was amplified with the primer pair FgAgo1-GFP-F/-R (Table S3). The resulting PCR products were co-transformed with *Xho*I-digested PYF11^61^ into XK1-25^62^. The FgAgo1-GFP fusion vector was recovered from yeast transformants and subsequently transformed into *Escherichia coli* DH5α. The recombined plasmid was extracted from DH5α and transformed into ∆FgAGO1. The FgDicer2-GFP fusion construct was conducted using the same strategy. For observation of GFP fusion protein localization, fresh mycelia and conidia were examined with the Zeiss LSM780 confocal microscope (Carl Zeiss AG, Germany).To observe nuclei, fresh mycelia or conidia were washed with sterilized water and stained with 10 μg mL^−1^ 4′6-diamidino-2-phenylindole (DAPI, Sigma).

### Pathogenicity assay

After incubation in CMC for 4 days, conidia of each strain were collected by filtration through three layers of gauze and subsequently re-suspended in sterile distilled water to a concentration of 1 × 10^5^ conidia ml^−1^. A 10 μl aliquot of conidial suspension was injected into a floret in the central section spikelet of single flowering wheat head of susceptible cultivar Jimai 22. There were ten replicates for each strain. After incubation at 22 ± 2°C under 95–100% humidity for 15 dats, the infected spikelets in each inoculated wheat head were recorded. To examine infection in tomato, a 10 μl aliquot of each conidial suspension was injected into the wounded tomato after surface sterilization. There were five replicates for each strain. Inoculated tomatoes were incubated at 25 °C and 100% humidity with 12 h of daylight, and were photographed 3 days after inoculation. The pathogenicity experiment was repeated three times.

### RNA isolation and quantitative RT-PCR (qRT-PCR)

To extract total RNA, mycelia of each strain were inoculated in potato dextrose broth (PDB) and cultured for 24 h at 25 °C in the dark. Mycelia were harvested by filtration over two layers of miracloth, washed with sterilized water. Harvested mycelia were then lyophilized and ground in liquid nitrogen. Total RNA was extracted from mycelia of each sample using the TaKaRaRNAiso Reagent, and 10 mg of each RNA sample was used for reverse transcription with RevertAid H Minus First Strand cDNA Synthesis Kit employing the oligo(dT)_18_ primer (Fermentas Life Sciences, Burlington, Canada). The expression levels of target genes were determined by quantitative real-time PCR with the primers listed in Table S3. For each sample, PCR amplifications with the primer pair actin-F/-R (Table S3) for the quantification of expression of actin gene were performed as a reference. The experiment was repeated three times independently.

### Cloning and sequencing of *F. graminearum* sRNAs

Total RNA was obtained from mycelia of the wild type and ∆FgDICER2 grown in PDB for 24 h at 25 °C. The small RNA fractions were extracted from 15% denaturing poly-acrylamide gel. Subsequently, a 5′adaptor and a 3′ adaptor were ligated to 15 μg small RNAs, and then the resulting short RNAs were converted to DNA by RT-PCR and sequenced on an Illumina-Solexa machine by BGI company (Shenzhen, China). The sRNA-Seq was repeated three times. The sequenced short reads data of the wild type and ΔFgDICER2 were deposited to BioProject at NCBI under accession number PRJNA253151 and PRJNA253153 respectively.

### Sequence analysis

*F. graminearum* genome sequence was downloaded from the BROAD INSTITUTE website ( http://www.broadinstitute.org/annotation/genome/Fusariumgroup/MultiHome.html). After removing adaptor contaminants from the raw reads, the remaining sRNA sequences were mapped to the *F. graminearum* genome using SOAP ( http://soap.genomics.org.cn). Only the sequences which perfectly matched the genome along their entire length were considered. By comparing small RNAs against these known non-coding RNAs (rRNA, tRNA, snRNA, and snoRNA) deposited at Rfam database^63^ using BLAST, small RNAs belonging to the “rRNA etc.” class were not included in data analysis. The prediction of *F. graminearum* milRNAs was carried out using an algorithm named “MIREAP” which could identify known miRNAs and novel candidates with canonical hairpin structure and sufficiently supported by sequencing data ( https://sourceforge.net/projects/mireap). In addition, target genes of the candidate milRNAs were identified with the miRanda program^64^.

## Additional Information

**How to cite this article**: Chen, Y. *et al.* Characterization of RNA silencing components in the plant pathogenic fungus *Fusarium graminearum. Sci. Rep.* **5**, 12500; doi: 10.1038/srep12500 (2015).

## Supplementary Material

Supplementary Information

## Figures and Tables

**Table 1:** Comparisons of small RNAs in the wild-type HN9-1 and ∆FgDICER2 in RNA-seq assays.

**Type**	**Unique_sRNAS**	**Percent**	**Total sRNAs**	**Percent**
Total_sRNAs	3096205	100.00%	56413871	100.00%
∆FgDICER2& HN9-1	491334	15.87%	53359316	94.59%
∆FgDICER2_specific	1221557	39.45%	1442041	2.56%
HN9-1-specific	1383314	44.68%	1612514	2.86%
