# Supplementary material for: Characterization of RNA silencing components in the plant pathogenic fungus Fusarium graminearum
Source: Sci Rep. 2015 Jul 27;5:12500. doi: 10.1038/srep12500 (PMC4515635; doi:10.1038/srep12500)
Supplement: Supplementary Information [file srep12500-s1.pdf]

Supplementary data

Characterization of RNA silencing components in the plant pathogenic fungus *Fusarium graminearum*

Yun Chen, Qixun Gao, Mengmeng Huang, Ye Liu, Zunyong Liu, Xin Liu, Zhonghua Ma*****

Institute of Biotechnology, Zhejiang University, Hangzhou, 310058, China

Table S1 Summary statistics of small RNA libraries from wild type HN9-1 and ΔFgDICER2 strain

Library type	Total number of sequences (≥17 bp)	Perfect match to genome (Total)	Percent %(Total)	Total number of Unique SRNAs	Perfect match to genome (Unique)	Percent %(Unique)
WT-repeat 1	5612806	4909980	87.48%	593053	370310	62.44%
WT-repeat 2	11007292	9932370	90.23%	979460	644015	65.75%
WT-repeat 3	11704949	10530124	89.96%	696061	468682	67.33%
ΔFgDICER2 - repeat 1	5370188	4680301	87.15%	473040	300763	63.58%
ΔFgDICER2 - repeat 2	11106651	10016054	90.18%	732155	486920	66.51%
ΔFgDICER2 - repeat 3	11611985	10407035	89.62%	926407	600928	64.87%

Table S2 Potential milRNA candidates in *F. graminearum*

milRNA	Mfe (kcal/mol)	milRNA_seq	Length	ΔFgDICER2 expression	HN9-1 expression	$\log_2$ (HN9-1/ΔFgDICER2)	p-value	Location	Targets
Fg-milRNA-1	-73	UCCGGUAUGGUGUAGUGGCUA	21	878367	715366	-0.01	2.26E-74	intergenic	NA
Fg-milRNA-2	-75.5	GAAUGUUGACCUCGGAUCAGG	21	0	105597	16.69	0	intergenic	NA
Fg-milRNA-3	-80.2	GGUACUGUGGUCUAGUUGGU	20	600	1003	0.68	2.55E-20	intergenic	FGSG_05319
Fg-milRNA-4	-73.2	UGGAGAAAAUUGAGAAGGCUCGA	23	0	892	13.96	9.02E-261	intergenic	FGSG_04063
Fg-milRNA-5	-66	UAGGAAAGGCAGUUAACUAGGA	22	0	794	10.92	7.21E-127	intergenic	NA
Fg-milRNA-6	-74.2	UUAUAUAUUUUUUGACUGUCA	21	0	64	7.29	1.58E-17	intergenic	NA
Fg-milRNA-7	-45.1	UAAACUGAGAAGAUUAGGGCU	21	0	43	6.72	1.18E-12	intergenic	NA
Fg-milRNA-8	-37.48	GACAAGGAGUGGUCGAGCGGUA	22	0	13	7.86	1.66E-04	intergenic	NA
Fg-milRNA-9	-25.6	AAGAAAAUGGGAGCGAGCACA	21	0	9	4.46	1.41E-03	intergenic	NA
Fg-milRNA-10	-83.5	GGAGGAGGAGAAGGAGGAGUC	21	0	9	7.33	2.43E-03	intergenic	FGSG_05213, 06912, 09782, 11546
Fg-milRNA-11	-89.7	AGGAUAUUAGUGGAUGGGUCGAG	23	0	8	4.29	2.87E-03	intergenic	NA
Fg-milRNA-12	-110.9	GGCAGCAUUGUUGACAGGGCCUU	23	168	0	-11.68	1.10E-54	intergenic	NA
Fg-milRNA-13	-37.48	GACAAGGAGUGGUCGAGCGGU	21	19	0	-4.96	1.90E-05	intergenic	NA
Fg-milRNA-14	-46.7	GAAACUGGAUGGCUUCGUAGA	21	17	0	-4.80	5.71E-05	intergenic	NA
Fg-milRNA-15	-77.7	UUGGUUGGGAACGUUGGUUA	20	14	0	-8.03	4.37E-05	intron_sense	FGSG_03308, 03713, 04480, 11026
Fg-milRNA-16	-37.2	CCGGGACGGUUUAACAUUCCCCUU	24	8	0	-7.22	3.19E-03	intergenic	NA
Fg-milRNA-17	-34.5	GACAACGUGGCCGAGUGGUU	20	545	0	-9.80	3.16E-85	intergenic	FGSG_07439
Fg-milRNA-18	-41.4	AGUGACAAGAAGAAGAACCGCC	22	14	0	-4.52	3.06E-04	exon_sense	NA
Fg-milRNA-19	-52.5	GAGGAAGAGGAGGAGGAGGAGGA	23	19	0	-4.96	1.90E-05	exon_sense	FGSG_12931,12883
Fg-milRNA-20	-28.9	UGCCAGAUCAUGCGAGACCCCCA	23	11	0	-4.17	1.71E-03	exon_sense	NA
Fg-milRNA-21	-53.1	AGGACUAGGGGGCGAAGCUCGG	22	39	0	-5.99	6.02E-10	intergenic	NA
Fg-milRNA-22	-33.4	AGCAAGGAGUGGUGUAGUGGGA	22	86	0	-7.14	2.75E-19	intergenic	NA
Fg-milRNA-23	-70.02	GGCGCAGUGGCAGAGUGGUCUA	22	672	905	0.72	4.63E-23	intergenic	NA

Fg-milRNA-24	-25.66	AAAGCGAUUCAAUUCAUCUUUU	22	12	0	-4.29	9.59E-04	intergenic	NA
Fg-milRNA-25	-27.2	GAUGAAGACUGAACCGUCGGGG	22	14	0	-4.52	3.06E-04	exon_sense	NA
Fg-milRNA-26	-29.58	GGCAACGUGGCGGAGUGGUU	20	2183	826	-1.11	3.32E-89	intergenic	FGSG_01703
Fg-milRNA-27	-64	UUGGGAUGAAUGGGCGGACAAUA	23	14	0	-4.52	3.06E-04	intron_sense	NA
Fg-milRNA-28	-61.86	AUGGGUACGGGCGGGUUGCGGAA	23	14	0	-4.52	3.06E-04	intergenic	NA
Fg-milRNA-29	-27.4	AGGAGGCAGUAAUAAGACUUG	21	18	0	-4.88	3.29E-05	intergenic	NA
Fg-milRNA-30	-28.7	CGCGACUGGGAUAAGACGACAA	22	14	0	-4.52	3.06E-04	exon_sense	NA
Fg-milRNA-31	-113.9	ACAUACGACCAUACCUACCAGA	22	497	0	-9.67	2.51E-79	exon_sense	NA
Fg-milRNA-32	-25.4	UGAGGACACUGCAUCACGACUCU	23	16	0	-4.71	9.96E-05	exon_sense	NA
Fg-milRNA-33	-41.17	GAUAAGGAGUGGUCGAGCGGGAU	23	14	0	-4.52	3.06E-04	intergenic	NA
Fg-milRNA-34	-60.62	GCGGGUUUAGCUCAGUUGGGAGA	23	0	533	10.35	8.30E-94	intergenic	NA
Fg-milRNA-35	-71.89	GGUGAGAUGGCCGAGUUGGU	20	0	1009	11.27	8.21E-152	intergenic	FGSG_11890,11505
Fg-milRNA-36	-30.2	GUGGGAUCUGAUGCACGAGAUGG	23	0	10	4.61	7.00E-04	exon_sense	NA
Fg-milRNA-37	-22	GACGAGACUGAGCGUAGGAA	20	0	8	4.29	2.87E-03	exon_sense	NA
Fg-milRNA-38	-38.8	CCUCUCGAUGACUCUGCGCAA	21	0	17	5.38	6.41E-06	exon_sense	NA
Fg-milRNA-39	-32.2	GGGGAUGGGUAUGGGUCGGGGC	22	0	9	4.46	1.41E-03	intergenic	NA
Fg-milRNA-40	-21.9	UCUGGAAGACUGAGUGGAUG	20	0	10	4.61	7.00E-04	exon_sense	FGSG_07238,05906
Fg-milRNA-41	-63.7	CUGGAUCAUUGUAGACCAGGCU	22	0	11	4.75	3.51E-04	exon_sense	NA
Fg-milRNA-42	-62.36	UGGGUACGGGCGGGUUGCGGA	21	0	11	4.75	3.51E-04	intergenic	FGSG_00248
Fg-milRNA-43	-27.9	UCUGCGGCGAUGAAUAGACACU	22	0	8	4.29	2.87E-03	intergenic	NA
Fg-milRNA-44	-61.4	GCUGUCGAGCGUGUAUGGGGUG	22	0	10	4.61	7.00E-04	intron_sense	NA
Fg-milRNA-45	-80.1	UGAUGUUUCAGAGGGCCGAGUUA	23	0	13	4.99	9.00E-05	exon_sense	NA
Fg-milRNA-46	-24.1	AGGACGUCUCGGAGCAGCAGA	21	0	10	4.61	7.00E-04	intron_sense	NA
Fg-milRNA-47	-72.1	AAGACUCAUAGAAAUUAGGGCA	22	0	15	5.20	2.37E-05	intergenic	NA
Fg-milRNA-48	-79.2	CGGGAAUUCGGGAAUUAGGGGGA	23	0	82	7.65	1.91E-21	intergenic	NA
Fg-milRNA-49	-96.9	GGCAACGUGACGGAGUGGUUA	21	6320	5713	0.15	2.82E-08	intergenic	NA

11

Table S3 PCR primers used in this study

Primer	**Sequence (5'-3')**	**Relevant characteristics**
FgRdRp1-KO1<br>FgRdRp1-KO2	ATctcgagTGCCAATACTTCACCTTCCA<br>ATgtcgacTGAAAGAGGCTGGACCAAAA	PCR primers to amplify *FgRdRp1* upstream fragment for the construction of *FgRdRp1* deletion mutants
FgRdRp1-KO3<br>FgRdRp1-KO4	ATaagcttTGCAACCAACTTACTTGGGT<br>ATggatccAAGCATCGTCGATTTGTGTCC	PCR primers to amplify *FgRdRp1* downstream fragment for the construction of *FgRdRp1* deletion mutants
FgRdRp1-IDF<br>FgRdRp1-IDR	CAACCTGCGACAGCATAGATG<br>TTAGCCTGGTCAACCAGCAGA	PCR primers for identification of *FgRdRp1* deletion transformants
FgRdRp2-KO1<br>FgRdRp2-KO2	ATctcgagTTTCCCATGGAGATCATGACC<br>ATgtcgacATGCGATGGCATACTAGCCAA	PCR primers to amplify *FgRdRp2* upstream fragment for the construction of *FgRdRp1* deletion mutants
FgRdRp2-KO3<br>FgRdRp2-KO4	ATaagcttAGCCTTTGCAGATGAATTGG<br>ATggatccCCATCGATCAAGTCGTTCAA	PCR primers to amplify *FgRdRp2* downstream fragment for the construction of *FgRdRp1* deletion mutants
FgRdRp2-IDF<br>FgRdRp2-IDR	GGCACGAGGATTCATTTGTCT<br>GCCATTTACGAGGTTGTTGA	PCR primers for identification of *FgRdRp2* deletion transformants
FgRdRp3-KO1<br>FgRdRp3-KO2	ATctcgagTGGAAGTCATCTGCCGAAAT<br>ATgtcgacTGCCTGACGTGAGAAGATTCT	PCR primers to amplify *FgRdRp3* upstream fragment for the construction of *FgRdRp1* deletion mutants
FgRdRp3-KO3<br>FgRdRp3-KO4	ATaagcttCGGCCTGTAAGTGGTATTGTT<br>ATggatccCGAATGCCATCTTCTGTACAA	PCR primers to amplify *FgRdRp3* downstream fragment for the construction of *FgRdRp1* deletion mutants
FgRdRp3-IDF<br>FgRdRp3-IDR	TGACACCATTTCATTTGACCA<br>TTTGATAGATGCGTCACCCT	PCR primers for identification of *FgRdRp3* deletion transformants
FgRdRp4-KO1<br>FgRdRp4-KO2	ATctcgagAGCGTATCGTACAACCAACCA<br>ATgtcgacTTCAGTGAAACCCCGTTCA	PCR primers to amplify *FgRdRp4* upstream fragment for the construction of *FgRdRp1* deletion mutants
FgRdRp4-KO3<br>FgRdRp4-KO4	ATggatccAGTTGGCTCCTAAGCGCTTT<br>ATgagctcTTATCCTCCGCTACCATTTGG	PCR primers to amplify *FgRdRp4* downstream fragment for the construction of *FgRdRp1* deletion mutants

FgRdRp4-IDF<br>FgRdRp4-IDR	AATTTGCCTCTGACAGATTCG<br>GAAATCAAAAATGTGGCACGG	PCR primers for identification of *FgRdRp4* deletion transformants
FgRdRp5-KO1<br>FgRdRp5-KO2	ATctcgagATGCCACCACCCAAAATAGA<br>ATgtcgacACCCAGAGCATGGAGAAGAAT	PCR primers to amplify *FgRdRp5* upstream fragment for the construction of *FgRdRp1* deletion mutants
FgRdRp5-KO3<br>FgRdRp5-KO4	ATggatccCGCCTCGTATACTTTGCCAAG<br>ATgagctcACCATCACAGTATACCTGGTA	PCR primers to amplify *FgRdRp5* downstream fragment for the construction of *FgRdRp1* deletion mutants
FgRdRp5-IDF<br>FgRdRp5-IDR	ATCTTATCTGCTGTGTAACAC<br>GTCCTGTCGCCCGAGCCCAGTC	PCR primers for identification of *FgRdRp5* deletion transformants
FgAGO1 -KO1<br>FgAGO1 -KO2	ATctcgagTTGGCCTTGCTCCACTAAGAA<br>ATgtcgacTAATAGCAGCATCGGGCTGA	PCR primers to amplify *FgAGO1* upstream fragment for the construction of *FgRdRp1* deletion mutants
FgAGO1-KO3<br>FgAGO1-KO4	ATggatccTTGGTACATCAGAGTCCGAAA<br>ATgagctcTACTTCGCTCGGAAGACCTC	PCR primers to amplify *FgAGO1* downstream fragment for the construction of *FgRdRp1* deletion mutants
FgAGO1-IDF<br>FgAGO1-IDR	ATGGGAGAGGACGAGGTGAT<br>CGTTGTGGAGCTTGTGGTTTA	PCR primers for identification of *FgAGO1* deletion transformants
FgAGO2-KO1<br>FgAGO2-KO2	ATctcgagATGTCTGATAGAGGGCGCTCA<br>ATgtcgacTCCATGCCAGTTTCTTACCGT	PCR primers to amplify *FgAGO2* upstream fragment for the construction of *FgRdRp1* deletion mutants
FgAGO2-KO3<br>FgAGO2-KO4	ATggatccTGTTCTTTCAGCATTCGCC<br>ATgagctcTAGCGAGATGCGCGTAGTAGA	PCR primers to amplify *FgAGO2* downstream fragment for the construction of *FgRdRp1* deletion mutants
FgAGO2-IDF<br>FgAGO2-IDR	TCGCCTAGCCCTGACAAGAT<br>ATGAGCCCACTCAACCAGCTT	PCR primers for identification of *FgAGO2* deletion transformants
FgDICER1-KO1<br>FgDICER1-KO2	ATctcgagACTCGACCGACAGCGAA<br>ATgtcgacAAAGCTGTGTCTTGGTCTCGA	PCR primers to amplify *FgDICER1* upstream fragment for the construction of *FgRdRp1* deletion mutants
FgDICER1-KO3<br>FgDICER1-KO4	ATggatccTCATCGACATGACCATCGTC<br>ATgagctcCCTTTGCAGCAGCAATCTTA	PCR primers to amplify *FgDICER1* downstream fragment for the construction of *FgRdRp1* deletion mutants
FgDICER1-IDF<br>FgDICER1-IDR	CGCAAATTGTCCGACGATAT<br>TCAAGTTCGGCCACATAGTCA	PCR primers for identification of *FgDICER1* deletion transformants

FgDICER2-KO1<br>FgDICER2-KO2	ATctcgagTGTCCTCAAGCGATAAGGTCA<br>ATgtcgacAAGGATTTCGTCGTGGATTG	PCR primers to amplify *FgDICER2* upstream fragment for the construction of *FgRdRp1* deletion mutants
FgDICER2-KO3<br>FgDICER2-KO4	ATggatccTTGGAGATGCGGTTCTTGACT<br>ATgagctcTCCATGGCAACAAGAAATCG	PCR primers to amplify *FgDICER2* downstream fragment for the construction of *FgRdRp1* deletion mutants
FgDICER2-IDF<br>FgDICER2-IDR	AGAATAGCTCTGGTCGAAAGG<br>TTTATTTCAACTTCAGGCCG	PCR primers for identification of *FgDICER2* deletion transformants
FgCYP51A-SP1<br>FgCYP51A-SP2	ATctcgagTGGTAGCACTCTTTGCTGTCA<br>ATaagcttGCTTTTTGCGTAAGGCCAAA	PCR primers to amplify the silencing target region (forward) of FgCYP51A gene for silencing vector construction
FgCYP51A-SP3<br>FgCYP51A-SP4	ATggtaccTGGTAGCACTCTTTGCTGTCA<br>ATagatctGCTTTTTGCGTAAGGCCAAA	PCR primers to amplify the silencing target region (reverse complement) of FgCYP51A gene for silencing vector construction
FgCYP51A-S-RTF<br>FgCYP51A-S-RTR	CCTGGACCCTTGTGGCTTCT<br>ACCTCTCGCTCGATTAACTGGAC	RT-PCR primers to quantify the mRNA expression level of FgCYP51A
FgPKS12-SP1<br>FgPKS12-SP2	ATctcgagACTTTGCCGACATTCAGGAA<br>ATaagcttAAGGAACTGACGATCGAGCAA	PCR primers to amplify the silencing target region (forward) of FgPKS12 gene for silencing vector construction
FgPKS12-SP3<br>FgPKS12-SP4	ATggtaccACTTTGCCGACATTCAGGAA<br>ATagatctAAGGAACTGACGATCGAGCAA	PCR primers to amplify the silencing target region (reverse complement) of FgPKS12 gene for silencing vector construction
FgCNB1-SP1<br>FgCNB1-SP2	ATGCctcgagGGACGCTCCCATCACCGTCTGTG<br>ATGCaagctt AAGGTGAAGTAGTCGGTCAAGT	PCR primers to amplify the silencing target region (forward) of FgCNB1 gene for silencing vector construction
FgCNB1-SP3<br>FgCNB1-SP4	ATGCggtaccGGACGCTCCCATCACCGTCTGTG<br>ATGCagatct AAGGTGAAGTAGTCGGTCAAGT	PCR primers to amplify the silencing target region (reverse complement) of FgCNB1 gene for silencing vector construction
FgCNB1-S-RTF<br>FgCNB1- S-RTR	CAGCATAGAGTGTGTTCTCTAC<br>CACAGAAAGAATCCATGCATGCCT	RT-PCR primers to quantify the mRNA expression level of FgCNB1
CPT-SP1<br>CPT-SP2	TACTCCGAAGAACCACTTGTT<br>TGACAGCAAAGAGTGCTACCACATTGTTGTCCTTCCTTGTCTT	PCR primers to amplify the silencing target region of FgTIR6 gene for CPT co-silencing vector pSilent-CPT construction

CPT-SP3<br>CPT-SP4	TGGTAGCACTCTTTGCTGTCA<br>GCTTTTTGCGTAAGGCCAAACT	PCR primers to amplify the silencing target region of FgCYP51A gene for CPT co-silencing vector pSilent-CPT construction
CPT-SP5<br>CPT-SP6	AGTTTGGCCTTACGCAAAAAGCCGTACCTTTCCTTCGGAGAAC<br>AGGAACTGACGATCGAGCAAGTC	PCR primers to amplify the silencing target region of FgPKS12 gene for CPT co-silencing vector pSilent-CPT construction
CPT-SP7<br>CPT-SP8	ATGCctcgagGCTTCGACTTTGACTTCGCGAAC<br>ATGCaagcttGAGAGCTCCAAGGACAAAGAAT	PCR primers to amplify the fusion target region (forward) of FgTRI6-PKS12-CNB1 for silencing vector pSilent-CPT construction
CPT-SP9<br>CPT-SP10	ATGCggtaccGCTTCGACTTTGACTTCGCGAAC<br>ATGCagatctGAGAGCTCCAAGGACAAAGAAT	PCR primers to amplify the fusion target region (reverse complement) of FgTRI6-PKS12-CNB1 for silencing vector pSilent-CPT construction
FgAGO1-RTF<br>FgAGO1-RTR	CACCAAGGCTGTGAGCATTT<br>TTGGGTCACTGGCACCTAAG	RT-PCR primers to quantify the mRNA expression level of FgAGO1
FgAGO2-RTF<br>FgAGO2-RTR	TTATTCGGGAGAAGCAAGCC<br>CAGCAGGAGTCTGTCCACAAGG	RT-PCR primers to quantify the mRNA expression level of FgAGO2
FgDICER1-RTF<br>FgDICER1-RTR	CTTGCCTCCTTTCGGACCTTTAC<br>TCTTGAGCGTTGAACATTGCTTT	RT-PCR primers to quantify the mRNA expression level of FgDICER1
FgDICER2-RTF<br>FgDICER2-RTR	GAGGAACTCGCCAAACTAGCA<br>CTTCTCTGCACGTTTCTGCTCC	RT-PCR primers to quantify the mRNA expression level of FgDICER2
FgAGO1-GFP-F<br>FgAGO1-GFP-R	TTTCGTAGGAACCCAATCTTCAAAATGGCGGACAGAGGTGATCGA<br>CACCACCCCGGTGAACAGCTCCTCGCCCTTGCTCACGATATAGTACATCGAGTTGGC	PCR primers to amplify the native promoter region and open reading frame of FgAgo1 for GFP fusion protein construction
FgAGO1-GFP-IDF<br>PYF11-GFP-IDR	TGCTACTTGTTTGGCCGAGCC<br>GACACGCTGAACTTGTGGCCGTT	PCR Primers for PYF11-FgAgo1-GFP plasmid identification
FgDICER2-GFP-F<br>FgDICER2-GFP-R	TTTCGTAGGAACCCAATCTTCAAAATGTCCTCAAGCGATAAGGTC<br>CACCACCCCGGTGAACAGCTCCTCGCCCTTGCTCACAATGAGTTCCATGGCAACAAG	PCR primers to amplify the native promoter region and open reading frame of FgDicer2 for GFP fusion protein construction

FgDICER2-GFP-IDF	GGCTGTTGATGGGCCTATAAAG	PCR Primer for PYF11-FgAgo1-GFP plasmid identification
PYF11-GFP-IDR	GACACGCTGAACTTGTGGCCGTT	PCR Primer for PYF11-FgAgo1-GFP plasmid identification
Fg05319-RTF	CAGCTCCCAAGACATGGTT	RT-PCR primers to quantify the mRNA expression level of
Fg05319-RTR	TAGAATCCTCCGCATCCAGA	FGSG_05319
Fg04063-RTF	TTGATGGAGAAGTCAGAAGGG	RT-PCR primers to quantify the mRNA expression level of
Fg04063-RTR	TCTTCTTCCACTCCGTTTCCT	FGSG_04063
hph-F	GGAGGTCAACACATCAATGCCTATT	PCR primers for amplification of hygromycin resistance gene
hph-R	CTACTCTATTCCTTTGCCCT	(HPH)
neo-F	GGAGGTCAACACATCAATGCT	PCR primers for amplification of neomycin resistance gene
neo-R	TTCAACACACAACAAATAAGA	(NEO)

Figure S1. Sequence analyses of the RNA silencing components in *F. graminearum*. (a) Predicted domains of RdRPs and argonaute and dicer proteins in *F. graminearum.* Lines represent the full length of proteins. Boxes represent the identified domains and are labeled with different colors. (b) Phylogenetic analyses of RdRps (left panel), argonaute proteins (middle), and dicer proteins (right panel) from *F. graminearum* (Fg) and other fungi, including *N. crassa* (NC), *M. grisea* (MG), *C. parasitica* (CP), *M. circinelloides* (MC), and *P. marneffei* (PM). The phylogenetic trees based on the domains were constructed using the maximum-likelihood method with 1000 bootstrap replicates. Counterparts from *Arabidopsis thaliana* were used as out-cluster controls. The amino acid sequences of RdRp homologues proteins: *N. crassa*, NC-qde1, (NCU07534); NC-sad1, (NCU0217); NC-rrp3, (NCU08435); *M. grisea*, MG1, (MG07682); MG2, (MG02748); MG3, (MG06205); *C. parasitica,* CP, (CCV01471); *M. circinelloides*, MC1, (EPB87370); MC2, (EPB93059.1); CN, *C. neoformans* (XP_571800); *A. thaliana*, AT1, (At4g11130); AT2 (At3g49500).The amino acid sequences of argonaute homologues proteins: *M. circinelloides,* MC-ago1,(EPB92043); MC-ago2,(EPB81851); MC-ago3,(EPB81974); *C. parasitica*, CP-ago1, (ACY36939); CP-ago2, (ACY36940); CP-ago3, (ACY36941); CP-ago4, (ACY36942); *C. neoformans*, CN1, (CNJ00490); CN2, (CNJ00610); *M. grisea*, MG1, (MG01294); MG2, (MG11029); MG3, (MG10003); *N. crassa*, NC-qde2, (NCU04730); NC-sms2, (NCU09434); *A. thaliana*, AT-ago1, (At1g48410); AT-ago2, (At1g31280); AT-ago3, (At1g31290); AT-ago4, (At2g27040); AT-ago5, (At2g27880); ATago6,(At2g32940); AT-ago7, (At1g69440); AT-ago8, (At5g21030); The amino acid sequences of dicer homologues proteins: *C. neoformans*, CN-dcl1, (CNC03670); CN-dcl2, (CNC03680); *M. grisea*, MG-dcl1 (MG01541); MG-dcl2, (MG07167); *N. crassa*, NC-dcl1, (NCU08270); NC-dcl2, (NCU06766); *A. thaliana*, AT-dcl1,

(At1g01040); AT-dcl2, (At3g0330); AT-dcl3, (At3g43920); AT-dcl4, (AT5G20320); *C. parasitica*, CP-dcl1(Q2VF19); CP-dcl2 (Q2VF18); *M. circinelloides,* MC-dcl1, (EPB86385); MC-dcl2, (CAZ65730); *P. marneffei*, PM-dcl1(KC686608); PM-dcl2, (KC686609).

Figure S2. Generation and identification of RNAi gene deletion mutants in *F. graminearum*. (a) PCR analyses for identification of single gene or double gene deletion mutant. PCR products were amplified with primers shown in table S1. The order of lane as followed by: M, 250 bp DNA ladder marker; lane 1, the wild type FgRdRp1, 2880 bp; lane 2, ΔFgRdRp1, 1900 bp; lane 3, the wild type FgRdRp2, 1990 bp; lane 4, ΔFgRdRp2, 1803 bp; lane 5, the wild type FgRdRp3, 2733 bp; lane 6, ΔFgRdRp3, 1873 bp; lane 7, the wild type FgRdRp4, 1900 bp; lane 8, ΔFgRdRp4, 1815 bp; lane 9, wild type FgRdRp5, 1870 bp; lane 10, ΔFgRdRp5, 1750 bp; lane 11, the wild type FgAGO1, 2477bp; lane 12, ΔFgAGO1, 1968 bp; lane 13, ΔFgAGO12, 1968 bp; lane 14, the wild type FgAGO2, 1452 bp; lane 15, ΔFgAGO2, 1755 bp; lane 16, ΔFgAGO12, 1350 bp;lane 17, the wild type FgDICER1, 3352 bp; lane 18, ΔFgDICER1, 2000 bp; lane 19, ΔFgDICER12, 1600 bp; lane 20, the wild type FgDICER2, 3165 bp; lane 21, ΔFgDICER2, 1870 bp; lane 22, ΔFgDICER12, 1870 bp. (b) Southern blot hybridization analyses of mutants using a 1349 bp *HPH* fragment as a probe. (c) Southern blot hybridization analyses of double-gene mutants ΔFgAGO12, ΔFgDICER12 using an 1100 bp *NEO* fragment as a probe.

Figure S3. The role of RNAi components in the growth, virulence, and stress response of *F. graminearum*. (a) Colony morphology of wild-type HN9-1 and RNAi-related gene deletion mutants. Photographs were taken after 5 days of incubation at 25°C on PDA. (b) Disease symptoms on wheat head and tomato caused

by wild-type HN9-1 and RNAi component deletion mutants. Wheat heads were point inoculated with the conidial suspension of each strain, and infected wheat heads were examined 15 days after inoculation. To test the virulence on tomato, we inoculated mycelia of the strains onto the surface of the tomato and incubated it at 25°C for 3 days. After incubation, the mycelia were scraped off for imaging. (c) Susceptibility of the wild-type HN9-1 and derivate mutants to various stress conditions. Serial dilutions of conidial suspension of each strain were spotted onto MM plates containing the indicated concentrations of methylmethane sulfonate (MMS), hydroxyurea, histidine, NaCl, KCl, glucose, sorbitol, Congo red, caffeine, $MgCl_2$, or $CaCl_2$. The plate photographs were taken after 2 days of incubation at 25°C.

Figure S4. Proposed RNAi pathway in *F. graminearum*. The pre-milRNA or hpRNA precursors are processed by FgDicer2, after which siRNA/ex-siRNA is loaded onto the RNA-induced silencing complex (RISC). FgAgo1 is a major component in the RISC. Mature milRNA or siRNAs target the cognate mRNA or genome locus for gene silencing. Amplification of the sRNA pool depends on FgRdRp proteins. FgDicer1 and FgAgo2 play minor or no roles in the milRNA- and hpRNA-induced gene silencing pathway in the mycelia of *F. graminearum.*

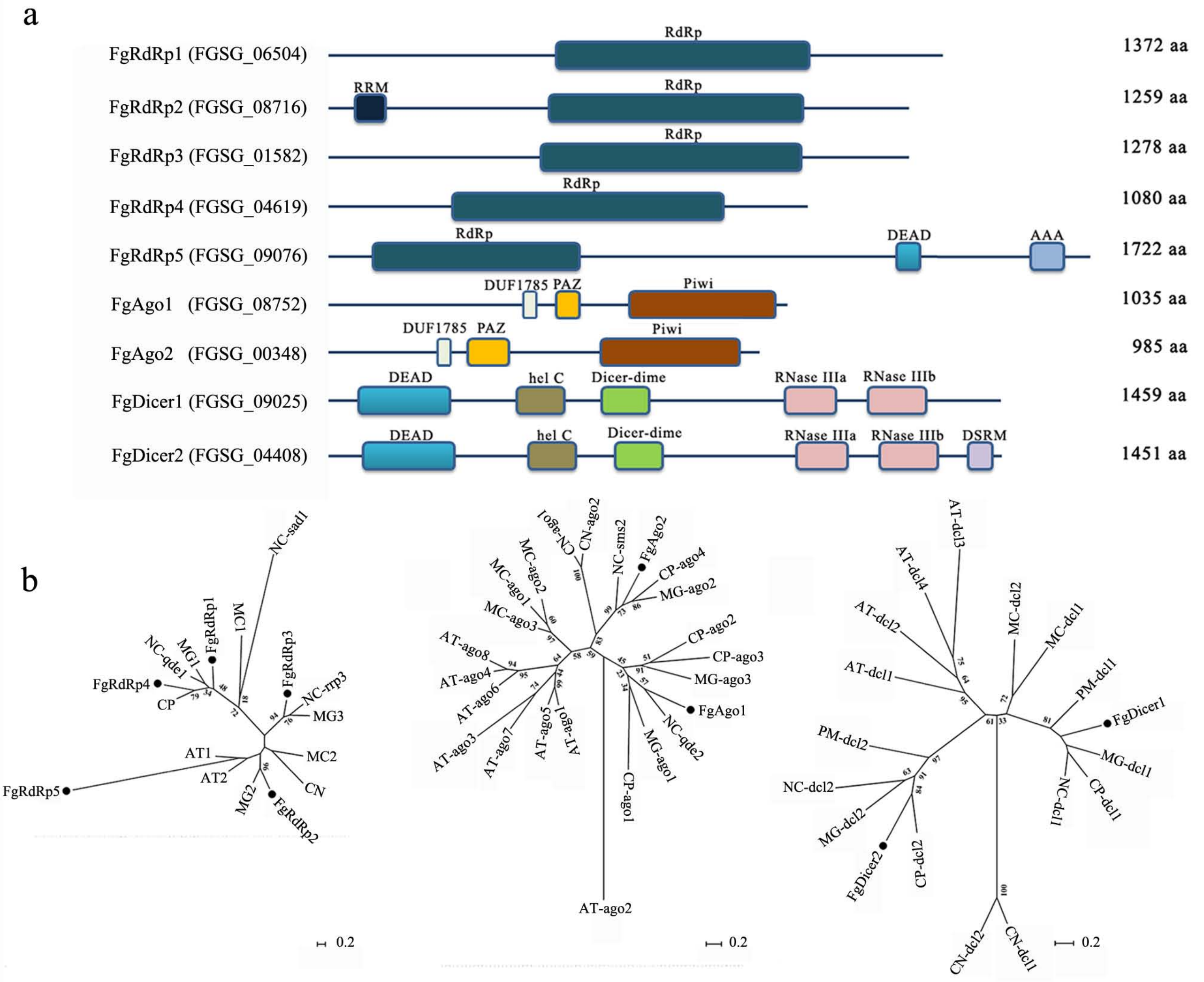
a
FgRdRp1 (FGSG_06504)
RdRp
1372 aa
FgRdRp2 (FGSG_08716)
RRM
RdRp
1259 aa
FgRdRp3 (FGSG_01582)
RdRp
1278 aa
FgRdRp4 (FGSG_04619)
RdRp
1080 aa
FgRdRp5 (FGSG_09076)
RdRp
DEAD
AAA
1722 aa
FgAgo1 (FGSG_08752)
DUF1785
PAZ
Piwi
1035 aa
FgAgo2 (FGSG_00348)
DUF1785
PAZ
Piwi
985 aa
FgDicer1 (FGSG_09025)
DEAD
hel C
Dicer-dime
RNase IIIa
RNase IIIb
1459 aa
FgDicer2 (FGSG_04408)
DEAD
hel C
Dicer-dime
RNase IIIa
RNase IIIb
DSRM
1451 aa
b
NC-sad1
MC1
FgRdRp1
MG1
NC-qde1
FgRdRp4
CP
FgRdRp3
NC-rrp3
MG3
MC2
CN
AT1
AT2
FgRdRp5
MG2
FgRdRp2
0.2
CN-ago1
CN-ago2
NC-sms2
FgAgo2
CP-ago4
MG-ago2
MC-ago2
MC-ago1
MC-ago3
AT-ago8
AT-ago4
AT-ago6
AT-ago3
AT-ago7
AT-ago5
AT-ago1
CP-ago2
CP-ago3
MG-ago3
FgAgo1
NC-qde2
MG-ago1
CP-ago1
AT-ago2
0.2
AT-dcl3
AT-dcl4
AT-dcl2
AT-dcl1
MC-dcl2
MC-dcl1
PM-dcl1
FgDicer1
MG-dcl1
CP-dcl1
NC-dcl1
PM-dcl2
NC-dcl2
MG-dcl2
FgDicer2
CP-dcl2
CN-dcl2
CN-dcl1
0.2

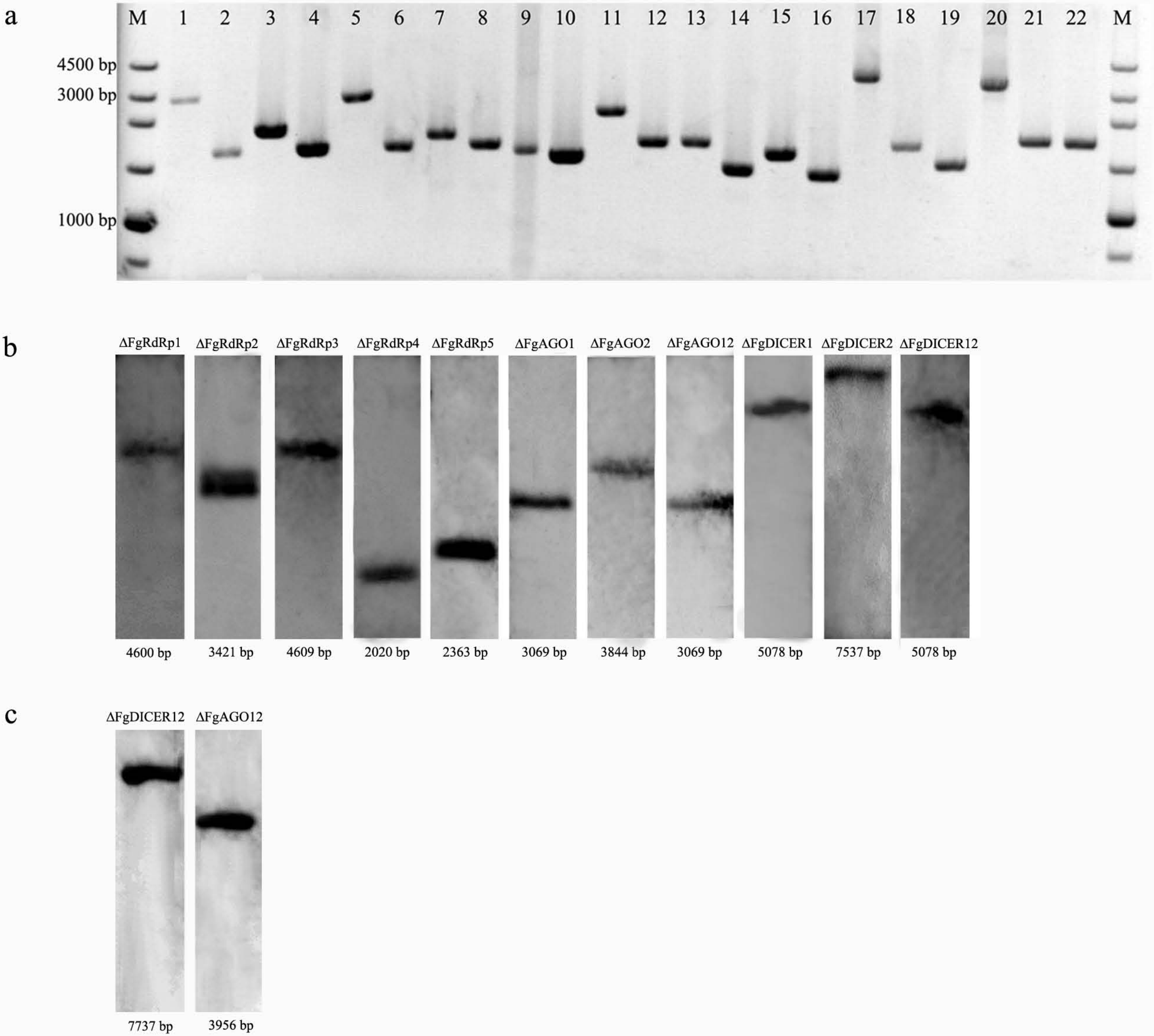
a
M 1 2 3 4 5 6 7 8 9 10 11 12 13 14 15 16 17 18 19 20 21 22 M
4500 bp
3000 bp
1000 bp
b
ΔFgRdRp1
ΔFgRdRp2
ΔFgRdRp3
ΔFgRdRp4
ΔFgRdRp5
ΔFgAGO1
ΔFgAGO2
ΔFgAGO12
ΔFgDICER1
ΔFgDICER2
ΔFgDICER12
4600 bp
3421 bp
4609 bp
2020 bp
2363 bp
3069 bp
3844 bp
3069 bp
5078 bp
7537 bp
5078 bp
c
ΔFgDICER12
ΔFgAGO12
7737 bp
3956 bp

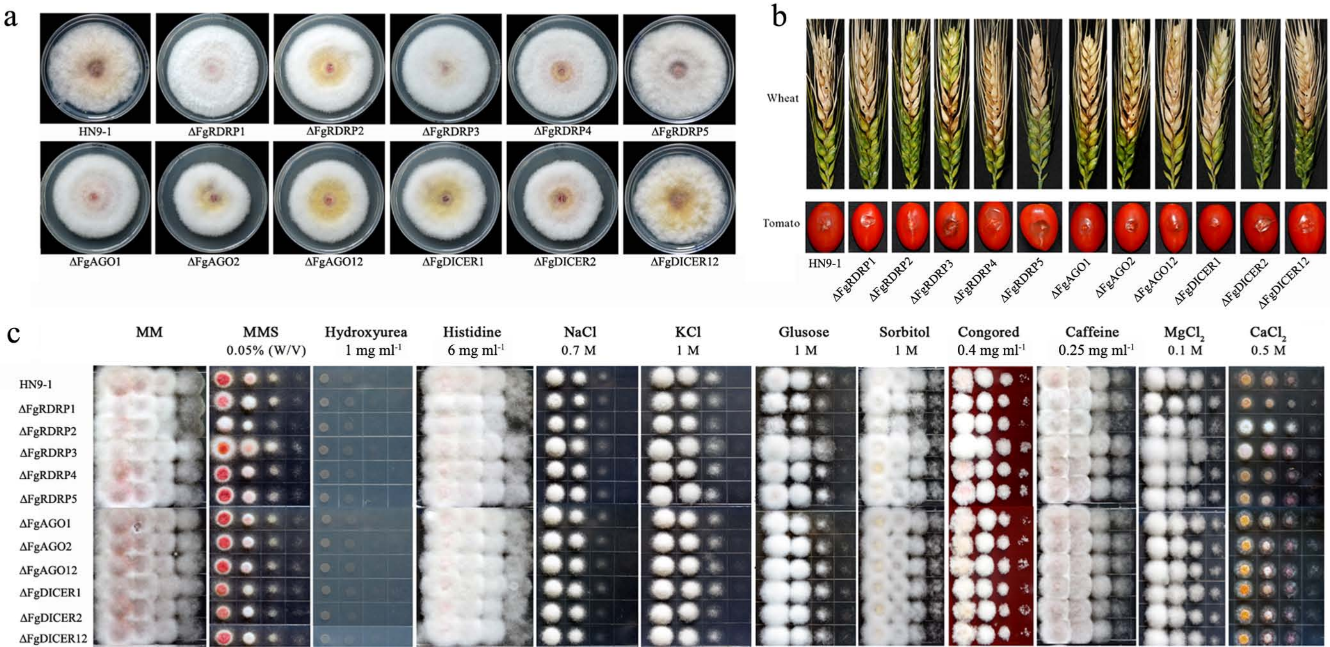

a
HN9-1
ΔFgRDRP1
ΔFgRDRP2
ΔFgRDRP3
ΔFgRDRP4
ΔFgRDRP5
ΔFgAGO1
ΔFgAGO2
ΔFgAGO12
ΔFgDICER1
ΔFgDICER2
ΔFgDICER12
b
Wheat
Tomato
HN9-1
ΔFgRDRP1
ΔFgRDRP2
ΔFgRDRP3
ΔFgRDRP4
ΔFgRDRP5
ΔFgAGO1
ΔFgAGO2
ΔFgAGO12
ΔFgDICER1
ΔFgDICER2
ΔFgDICER12
c
MM
MMS 0.05% (W/V)
Hydroxyurea 1 mg ml-1
Histidine 6 mg ml-1
NaCl 0.7 M
KCl 1 M
Glusose 1 M
Sorbitol 1 M
Congored 0.4 mg ml-1
Caffeine 0.25 mg ml-1
MgCl2 0.1 M
CaCl2 0.5 M
HN9-1
ΔFgRDRP1
ΔFgRDRP2
ΔFgRDRP3
ΔFgRDRP4
ΔFgRDRP5
ΔFgAGO1
ΔFgAGO2
ΔFgAGO12
ΔFgDICER1
ΔFgDICER2
ΔFgDICER12

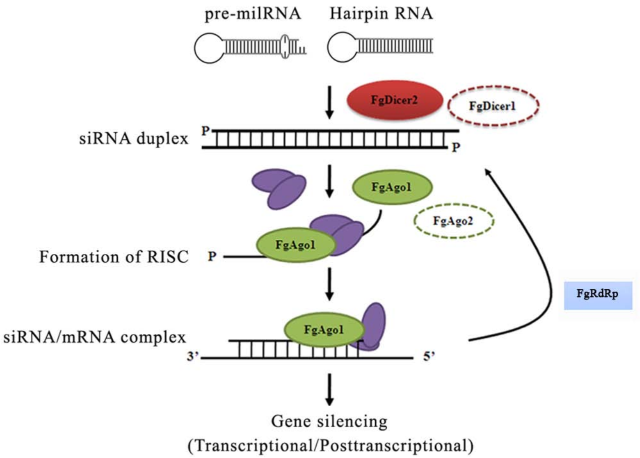
pre-milRNA
Hairpin RNA
FgDicer2
FgDicer1
siRNA duplex
P
P
FgAgo1
FgAgo2
Formation of RISC
P
FgAgo1
FgRdRp
siRNA/mRNA complex
FgAgo1
3'
5'
Gene silencing
(Transcriptional/Posttranscriptional)
